# Depression as a Failed Anxiety: The Continuum of Precision-Weighting Dysregulation in Affective Disorders

**DOI:** 10.3389/fpsyg.2021.657738

**Published:** 2021-07-22

**Authors:** Valery Krupnik

**Affiliations:** Naval Hospital Camp Pendleton, Camp Pendleton, CA, United States

**Keywords:** depression, anxiety, allostasis, interoception, predictive processing, precision-weighting, stress response

## Abstract

Depressive, anxiety, and trauma-related disorders have many symptoms in common such as unstable mood, high anxiety, sleep disturbance, impaired concentration among others. This degeneracy creates ambiguity in classifying psychiatric disorders and raises the question of their categorical vs. dimensional nature. Consequently, such ambiguity presents a dilemma for choosing diagnosis-specific vs. trans-diagnostic therapies. In this paper, I build on a theory that considers affective disorders on the continuum of stress response from normative to traumatic. Using an integrative evolutionary-stress response-predictive processing (iESP) model, I arrange affective disorders on a continuum of precision-weighting dysregulation, where depressive, anxiety and trauma-induced disorders have a characteristic pattern of precision-weighting dysregulation. I specifically address the relationship between anxiety and depressive stress responses, exploring the role of anxiety in the dynamics of depressive stress response and the resulting high co-occurrence of anxiety and depression symptoms. Finally, I discuss the model's relevance for therapy of depression.

The high comorbidity of anxiety and depressive disorders has been consistently demonstrated in multiple studies (Kessler et al., 2015). The meaning of this comorbidity remains a matter of contention. An important distinction has been made between epidemiologic comorbidity (over life-time) and clinical comorbidity, co-occurrence at the same time of two or more disorders (Kraemer et al., 2006), where epidemiologic comorbidity shows higher rates than clinical. This distinction raises the question of whether clinical comorbidity represents co-occurrence of distinct syndromes or distinct manifestations of the same syndrome.

This ambiguity has been reflected in the notion of *anxious depression* (Gersh and Fowles, 1979). In the DSM-5 (*Diagnostic and Statistical Manual of Mental Disorder*, 5th ed.; *DSM-5*; American Psychiatric Association, 2013), the “anxious distress” specifier has been added to the major depression diagnosis defined as the presence of at least two of the following five symptoms during a major depressive episode: (1) feeling keyed up or tense, (2) feeling unusually restless, (3) difficulty concentrating because of worry, (4) fear that something awful may happen, and (5) feeling that the individual might lose control of themselves. Several models have been offered to explain the frequent co-occurrence of anxiety and depression (for review see Mineka et al., 1998; Watson, 2009; Cummings et al., 2014). They include shared risk factors, causal relationship in both directions, and a dimensional view of anxiety and depressive disorders. Co-occurrence of anxiety and depressive symptoms raises the question of therapeutic strategy, i.e., how specific to the symptoms, if at all, should interventions be, and should they be specific, which symptoms would be a priority target? Despite the ever-growing number of treatments for depressive disorders no progress in outcome has been made since 1980 through 2010 (Cuijpers et al., 2011), and “anxious depression” has a poorer response to treatment with symptoms of anxiety and somatization showing higher resistance to antidepressants (Fava et al., 2008; Brown et al., 2016).

In this paper, I elaborate an integrative Evolutionary-Stress Response-Predictive Processing (iESP) model of depression (Krupnik, 2020a). The model describes depression as a normative (in the evolutionary view) depressive stress response arrested midway due to malfunction of predictive processing, more specifically, dysregulation of precision-weighting. I present affective disorders as complementary adaptive stress responses on the continuum of stress and then describe the depressive stress response as a dynamic process with a transient phase of anxiety. Next, I present anxiety, depressive, and traumatic stress responses in the predictive processing framework, placing them on the continuum of precision-weighting dysregulation. I elaborate the notion of specificity and relativity in relation to precision. Finally, I discuss how the model can instruct therapy for depression.

## Depression, Anxiety, and Trauma on The Continuum of Stress

The concept of allostasis is central to the discourse about stress and stress response. Environmental disturbance can drive the organism away from its homeostatic state. In case of failure to return to homeostasis under the pressure from environmental stressors, the organism undergoes allostasis (“stability through change”) (Sterling and Eyer, 1988; McEwen and Wingfield, 2003) to a new, sub-optimal, homeostatic state which may lead to pathology. In such an outcome, the organism experiences allostatic overload. A distinction has been made between type 1 and 2 allostatic overload (McEwen and Wingfield, 2003). Type 1 can be viewed as a situation where a disturbance overwhelms the organism's coping resources, triggering an emergency response curtailing functions non-essential for immediate survival. Type 2 refers to the situation of chronic stress that creates a drift away from the initial homeostatic state without triggering gradual drigt an emergency response. In a recent model of the stress continuum, stress response is subdivided into normative, pathogenic, and traumatic stress responses (Krupnik, 2019b, 2020b)^1^. Normative stress response (NSR) is defined as a response to an environmental disturbance where the organism returns to the initial homeostatic state. In pathogenic stress response (PSR), the organism experiences allostatic overload type 2 and undergoes allostasis to a new sub-optimal homeostatic state, whereas in traumatic stress response (TSR), the organism experiences allostatic overload type 1 with the ensuing breakdown of self-regulatory functions and a transition to a PTSD-like state. The distinction between PSR and TSR was suggested to reflect the difference between adversity and trauma (Krupnik, 2019b, 2020b). In this model, a stress reaction with prolonged high anxiety or depression falls within PSR.

In evolutionary view, stress response is an adaptation to the inherent volatility of environments. Evolutionary theory of depression considers it an adaptation to insurmountable adversity (Nesse, 1999). Different aspects of this theory are focused on various facets of such adversity including social defeat, entrapment (Gilbert and Allan, 1998), loss and abandonment (Bowlby, 1980; Watt and Panksepp, 2009). In a recent conceptualization, these aspects are viewed under the general umbrella of failure, i.e., failure to meet the organism's needs. Such concept of *failure* encompasses the above aspects of *loss, entrapment*, and *defeat*, and is captured in the acronym FLED (Krupnik, 2020a). Likewise, anxiety may be considered an alternative adaptation to adversity, where the adversity is perceived as avoidable as, for example, in the “smoke detector principle” (Nesse, 1999). Thus, ASR prepares the organism to use its resources for compensatory behaviors meant to mitigate the overload, whereas DSR prepares the organism to conserve its resources through emotional and behavioral withdrawal meant to “ride the overload out.” Following this theory, an adversity that persists to the point of being perceived as insurmountable would trigger an ASR to DSR transition.

## Anxiety into Depression Transition

It has long been known that anxiety is more likely to precede depression than the other way around and a causal link was suggested for the sequential comorbidity from anxiety to depression with anxiety response style as a moderator (Starr et al., 2016). It also appears that depression may inhibit the consequent anxiety (Lavigne et al., 2015). However, the notion of comorbidity implies that anxiety and depression are separate syndromes. This may be true for some anxiety disorders, but there is no compelling reason to believe that a depressive disorder can develop without any (even transient) increase in anxiety.

Unlike the categorical/diagnostic view of depression, evolutionary theory considers it not a state of mind but a dynamic process. For example, in an early animal model (Kaufman and Rosenblum, 1967), depression is viewed as a progression from an agitated state of protest to withdrawal and then recovery. The transition from protest to resignation was proposed as a core dynamic of depression, “This sequence from protest to despair provides a powerful animal model of human clinical depression,” (Zellner et al., 2011, p. 2). The protest stage of DSR implies an anxious reaction to the initial failure (FLED) where it is perceived as avoidable, and only when this perception turns to despair, the depressive stage of the DSR develops. Therefore, anxiety can be considered part of depression. Then, the term “anxious depression” denotes a depressive response either arrested at the protest stage or vacillating between protest and withdrawal. Consistent with this view is an observation that the early effect of SSRI antidepressants is downregulation of the amygdala response to emotional stimuli (Murphy et al., 2018).

From the standpoint of agency, the progression from protest to despair indicates a switch from the disposition of compromised self-efficacy to self-inefficacy. A central role of self-efficacy in depression and anxiety was suggested by the author of the self-efficacy concept over two decades ago (Bandura, 1988; Bandura et al., 1999). The influential learned helplessness theory of depression also points at self-inefficacy as a mediator of depressive response (Seligman, 1972). More recently, the role of self-efficacy in depression has been revisited in the predictive processing (Stephan et al., 2016) and iESP (Krupnik, 2020a) frameworks.

## Psychopathology As False Inference

Psychopathology has been increasingly interpreted in the predictive processing (PP) framework. PP no longer considers the brain as a passive acceptor and processor of sensory information (the “stimulus-cognition-response” paradigm) but as a predictive coding machine (Clark, 2013b; Friston et al., 2014). In PP, the brain's function is to guide the organism's response to environmental demands in the most adaptive, metabolically effective way, which is best accomplished by accurately predicting and preparing the organism for environmental challenges. States of the environment are not directly accessible to the brain but reflected in sensory input: exteroceptive for the external and interoceptive for the internal environments. By inferring the causes of sensations, the brain creates and updates a generative model of its environment which, in turn, allows for prediction of the incoming sensory stimuli. For example, if our brain infers that fire causes the burning sensation, we can predict (and prepare for) heat just from a sight of fire.

The brain's generative model is thought to operate according to Bayesian inferential statistics, where predictions (also referred to as inferences or hypotheses) about the states of the environment are represented not by fixed values but conditional probability distributions (Knill and Pouget, 2004; Friston, 2009; Clark, 2013a). To accurately model the inherently noisy environment, the brain's generative model biases and narrows down the probability distributions based on prior learning or prior beliefs, also called *priors*. In PP, prior beliefs are meant in a broad sense encompassing all cognitive levels from unconscious expectancies to declarative beliefs.

The mechanism of updating a generative model relies on *prediction error* (PE). When sensory input does not match the prediction (e.g., cool flame), a PE is generated. To maintain its accuracy, the generative model has to resolve the PE, which can happen in either of two ways: (a) through *posterior learning* by adjusting the model's prior into a posterior to better match the input (e.g., learning that certain chemicals can generate a low temperature flame), or (b) through *active inference* by adjusting the organism's properties and/or behavior so that it controls the sensory input in a way that matches the model's prediction (e.g., generating an illusion/hallucination of a burning sensation on contact with a cool flame). Through iterative cycles of perception-action, the brain directs the organism to selectively seek and gate sensory information to refine and fulfill its predictions.

The brain's generative model is thought to be embodied in its synaptic architecture and strength and organized hierarchically (Parr and Friston, 2018). Predictions are passed from top-down, originating in the higher cortical levels and descending through several relays to the sensory and motor areas, whereas sensory information passes in the opposite direction. At each relay, PE units compare predictions to the sensory input and generate PE that is signaled upward.

More recently, PP has been integrated with the free-energy principle (FEP) (Friston et al., 2006; Friston, 2010). According to it, an organism's generative model is continuously increasing its accuracy by minimizing its variational free energy (informational entropy). Such minimization is achieved by suppressing the cumulative PE. Variational free energy is defined as the upper limit on surprisal or *uncertainty* about the brain's sensory states. FEP stipulates that the brain's meta-function is to minimize its variational free energy, to which all brain functions can ultimately be traced. Thus, FEP has been suggested as a unified theory of the brain (Friston, 2010).

Minimization of free energy can sometimes be achieved through false inference. Illusions are a common example of false inference where PE is suppressed through adjusting perception to match the prior at the expense of veridicality, e.g., in the hollow-face illusion (Shipp et al., 2013). The idea of psychopathology as false inference has attracted much attention in clinical research, offering a universal explanation for the etiology of psychiatric conditions (Friston et al., 2014).

## Psychopathology As Dysregulation of Precision-Weighting

The notion of false inference is intrinsically connected to that of precision. To reflect states of the environment accurately, a generative model has to optimally weight its priors against PEs, which is known as *precision estimate or weighting* (Friston, 2009; Clark, 2013a). Priors and PEs are defined as probability distributions whose inverse variance is called *precision*. Dysregulated precision-weighting may manifest in too rigid (hyper-precise) priors refractory to updating by PE (underweighting of PE) or too accommodating (hypo-precise) priors (overweighting of PE). The former would render the generative model inflexible and unresponsive to environmental cues, whereas the latter—hyper-reactive to noise/random contingencies and thus lacking in predictive power. Precision-weighting is thought to be mediated by neuromodulatory control of the synaptic gain of PE units (Friston et al., 2014). Imbalance of precision-weighting may result in false/inaccurate inference and entail psychopathology, as has been proposed for depressive, anxiety, and trauma-related disorders.

## Depression, Anxiety, and Trauma on The Continuum of Precision-Weighting Dysregulation

Dysregulation of precision-weighting in depression has been approached from different angles of PP. From the standpoint of exteroception, overweighting of (“negative”) social exteroceptive prediction errors (ePEs) is viewed as depressogenic because over time it may create a negatively biased generative model of the social environment (Badcock et al., 2017). Consistent overweighting of negative social ePEs can lead to a biased set of posterior beliefs similar to Beck's depressive triad (Beck et al., 1979). From the standpoint of interoception, depression can be viewed as a disorder of allostasis, where the interoceptive generative model predicts a condition of chronic stress/allostatic overload type 2 (Barrett et al., 2016). In so doing, it underweights interoceptive PEs (iPEs) “locking the brain in” a perpetual stress response (Barrett et al., 2016; Badcock et al., 2017) and a state of selective interoceptive blindness (Krupnik, 2020a) even after the stressors relent. However, the possibility of overweighted iPEs in depression has also been noted (Clark et al., 2018), which contradiction will be addressed in the next section.

The role of higher-order priors in depression has also been proposed. Moutoussis and colleagues (Moutoussis et al., 2014) discuss the role of second-order priors in the efficacy of one's social interactions. Expected inefficacy may introduce a negative bias to the depressive generative model which then can be confirmed through active inference (by avoiding action or self-sabotage), leading to further overweighting of the “inefficacy” prior's precision. Allostatic self-efficacy was implicated in depression and fatigue as a meta-prior (Stephan et al., 2016). Predicting the range of allostasis, the self-efficacy prior can modulate the precision of lower-level allostatic priors, thus biasing the organism's stress response, e.g., enacting an energy conserving policy by decreasing the rate of metabolism in depression and fatigue.

Anxiety, unlike depression, is thought to be associated with hyper-precise exteroceptive priors insensitive to ePEs (Paulus et al., 2019). Such dysregulation creates a chronic prediction of threat from the environment. Due to the priors' rigidity, ePEs are minimized through active inference either by attentional bias toward threatening sensory signals or behavioral avoidance of uncertain/ambiguous situations. The interoceptive corollary to an anxious generative model is that the organism constantly predicts stress response, which prediction is confirmed by the active inference of high arousal and anxiety. That makes the model prone to misread interoceptive cues (“somatic errors”), thus underweighting iEPs (Paulus et al., 2019). In support of this view, anxiety was found to positively correlate with the discrepancy between actual and presumed interoceptive accuracy, where an overweighted belief in one's interoceptive accuracy predicted symptoms of anxiety (Garfinkel et al., 2016).

A contrast has recently been drawn between precision-weighting dysregulation in the depressive (DSR) vs. traumatic (TSR) stress response (Krupnik, 2020b). It was proposed that unlike the depressive generative model where iPEs are underweighted in the context of overweighted ePEs, the traumatic generative model underweights both iPEs and ePEs. The notion of hyper-precise exteroceptive priors in TSR has been formulated before (Wilkinson et al., 2017; Linson and Friston, 2019) and appears to be a consensus.

**Figure 1:**
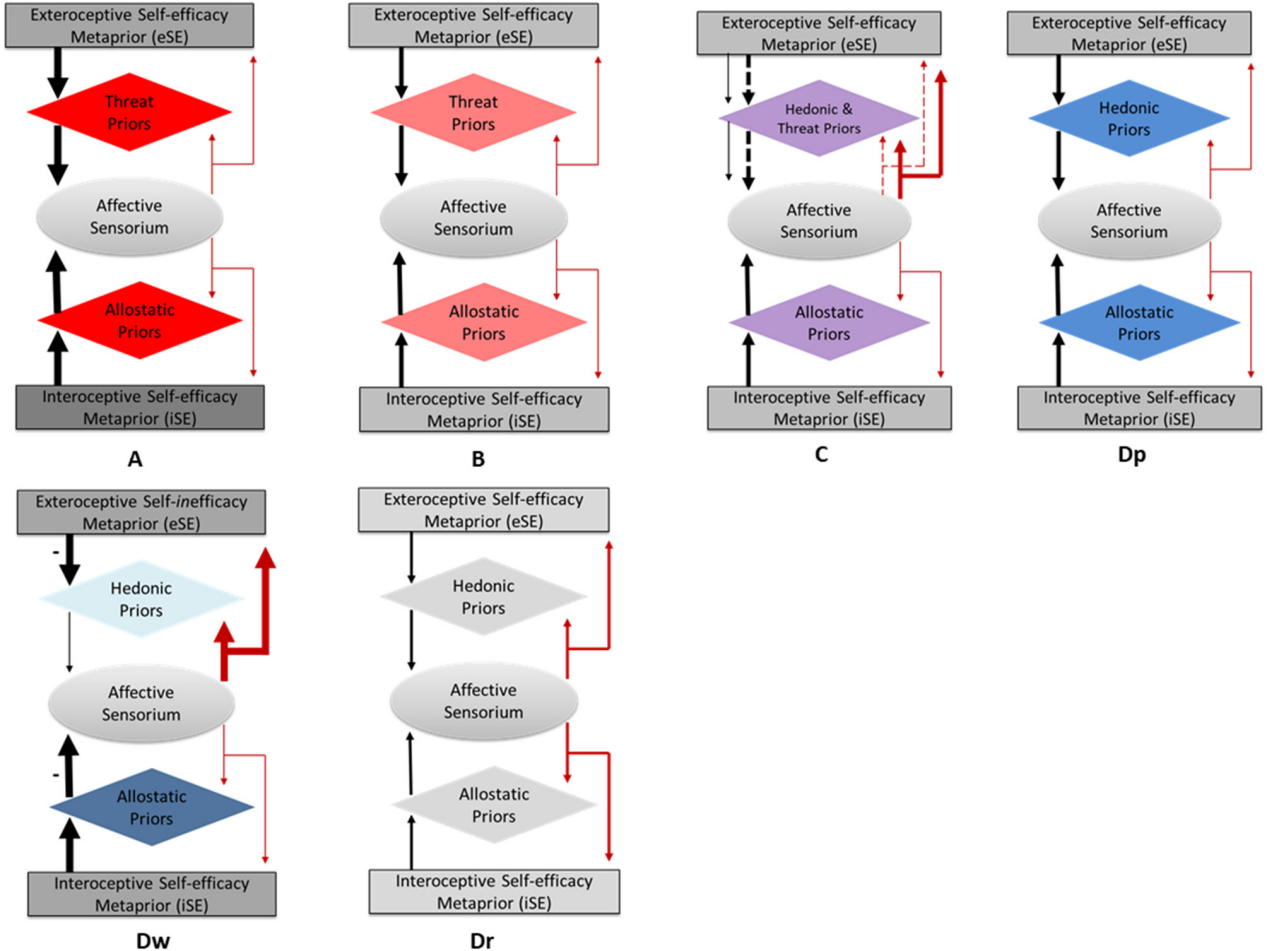
The continuum of precision-weighting dysregulation in affective disorders. **(A)** Traumatic stress response, **(B)** anxiety stress response, **(C)** mixed, anxiety-depressive stress response, **(Dp)** the protest stage of depressive stress response, **(Dw)** the withdrawal stage of depressive stress response, **(Dr)** the recovery stage of depressive stress response. The oval boxes represent affective sensorium including all affectively valenced sensations, both extero- and interoceptive. The rhombic boxes represent priors. The rectangular boxes represent self-efficacy metapriors. The intensity of the box's hue reflects the precision of the corresponding priors, and the thickness of arrows reflects the precision of the corresponding predictions. Black arrows represent predictions; red arrows represent prediction errors. The arrows point in the direction of information passaging. **(C)** The dashed arrows represent thereat-related predictions and prediction errors; the solid arrows represent hedonic predictions and prediction errors. **(Dw)** The minus sign by the downward arrow indicates the negative value of the self-efficacy metaprior, i.e., self-*in*efficacy. The minus side by the upward arrow indicates energy-conserving predictions (as opposed to energy expenditure predictions in the other modules).

In the DSM classification, depressive, anxiety, and trauma-related disorders belong in different categories (DSM-5), whereas in PP, they can be viewed as variations of precision-weighting dysregulation as captured in Figures 1A–D. In the next section, I discuss how these variations may relate to each other in the sense of co-existing, alternating, or morphing into one another.

## Precision-Weighting Dysregulation in iESP Model

Precision-weighting and its dysregulation are central to iESP (Krupnik, 2020a). R*elativity* and *specificity* are important aspects of precision-weighting. By under- or overweighted PEs I mean that their precision is under- or overweighted relative to the corresponding prediction which, in turn, is determined by the precision of the prior on which it is based (Figure 1). Therefore, a hyper-precise prior is tautological with hypo-precision of the corresponding PE (and vice versa), which makes people see a convex face in a concave image in the hollow-face illusion (Shipp et al., 2013) or might prevent one touching a cool flame even after seeing others do it. Precision-weighting is also specific in that precision of certain PE categories, as in perception of human faces, may not generalize to unrelated categories, for example, images of other concave objects do not necessarily produce a strong convex illusion.

In Figure 1, I present a model of the continuum of precision-weighting dysregulation in affective disorders stemming from depressive, anxiety, and traumatic stress responses (DSR, ASR, and TSR, respectively). The model was initially proposed for DSR and TSR (Krupnik, 2020a,b, 2021). In it, the main feature of DSR is considered overweighted ePEs (Figures 1C,Dp,Dw), specifically hedonic ones. From the phylogenetic standpoint, there are two primal behaviors, approach and avoidance, therefore corresponding sets of priors can be defined as hedonic and threat (Figure 1). The depressive generative model perceives the world as uncertain and unmanageable, and to minimize the free energy, its hedonic priors assume low precision with the entailing low precision of predicting/expecting pleasure, thus accommodating (and suppressing) ePEs (noxious distressing stimuli from the world) whose precision becomes relatively overweighted. The ensuing active inference biases the model toward sensory information associated with negative feeling states and makes it “overlook” the sensory input associated with positive feeling states thus underweighting “positive” ePEs. Hohwy (2013) suggests that given competing hypotheses about the world (e.g., it is generally agreeable vs. noxious), a generative model will choose the one with the lower cumulative ePE for the higher-level conscious representation, thus minimizing its free energy. Once settled on a negative worldview, the depressive generative model becomes further biased by selective attention guiding its active inference to selectively sample the world to confirm the bias, which will further minimize its free energy. The role of attention in the negatively biased depressive generative model has recently been explored and highlighted (Kube et al., 2020). On the interoceptive side, perceiving one's environment as uncertain and unmanageable may result in a prediction of allostatic overload. If this prediction becomes chronic, the allostatic priors may grow hyper-precise in the belief that life is inherently overbearing, which would trigger the active inference of energy conservation and negative emotions^2^. Of note, the magnitude of active inference may be indicative of the prior's precision, which could be useful for diagnostic purposes since priors' precision cannot be observed directly.

Another recently suggested regulator of the depressive generative model is self-efficacy (Figure 1). In PP, it is conceptualized as a meta-prior belief in the organism's ability to cope with stress (Stephan et al., 2016; Krupnik, 2020a), or in allostatic terms, the organism's trust in its range of allostasis. Stephan et al. (2016) suggest that prolonged psychological stress entailing dyshomeostasis leads to reduced self-efficacy. In turn, a low self-efficacy prior is fulfilled by active inference through avoiding active coping as in the “learned helplessness” model of depression (Seligman, 1972). To avoid confusion, I want to emphasize that high or low self-efficacy does not equal its high and low precision. For example, low self-efficacy may predict with high precision the organism's inability to withstand the allostatic load, driving the downstream predictions and enactment of energy-conserving policies of fatigue and depression (Figure 1Dw). Such a state may be better conceptualized as high-precision *self-inefficacy*. Likewise, high self-efficacy can have relatively low precision if it predicts effective coping in a narrow allostatic range^3^.

In iESP model (Krupnik, 2020a), self-efficacy serves as a master regulator of precision-weighting in stress response (Figure 1). Exteroceptive self-efficacy (eSE) issues top-down predictions about the organism's ability to respond to external challenges. Such predictions affect the precision of lower-level predictions about the challenge's outcome. For example, the sight of a fire may activate a high-precision eSE prediction that one can keep comfortably warm from an optimal distance. Such eSE may increase the precision of predicting comfort in the fire's vicinity, which could then be confirmed by the corresponding actions. If eSE's precision is low, it may depress the precision of predicting comfort, and the active inference may be delayed, leading to indecisiveness up to behavioral paralysis. I hypothesize that SE itself is organized hierarchically, where generalized/trait SE sits atop the hierarchy (corresponding to the psychological concept of self-confidence) regulating domain-specific SE (e.g., kinesthetic, social, intellectual) which, in turn, regulates context-specific SE. From this perspective, the meaning of personal development can be conceived of as maturation of the generative model driven by ever-increasing SE's precision. I suggest that the same logic can be applied to interoceptive SE (iSE). In the above example, iSE's precision may determine whether the organism will mobilize its energy for action or conserve it in a “freeze” response. Whether eSE and iSE can affect each other directly through a reciprocal neural connection is an open question. I am unaware of any evidence one way or the other, which is why they are shown without a direct link in Figure 1.

A fundamental property of iESP is that it is a dynamic model that considers stress response and psychopathology as a process, not a state. For DSR, it follows the aforementioned evolutionary view of depression as the sequence from protest to withdrawal and then recovery. The protest stage is where the eSE meta-prior (Figure 1Dp) still has its pre-depressive precision, sustaining the hedonic priors in face of adversity (FLED), thus underweighting “noxious” ePEs. Overtly, this may manifest cognitively in denial or disbelief and behaviorally in an attempt to compensate for or escape the FLED. Once such an attempt fails, and the FLED-driven ePEs continue propagating up the cognitive hierarchy, they may engage more attention thus increasing in precision, which would lead to increased free energy. To decrease it, the generative model may “relax” eSE's precision, leading to a decrease of the hedonic priors' precision down the hierarchy. Consequently, ePEs will be gaining in weight, driving eSE toward high-precision self-inefficacy (Figure 1Dw). In parallel, the increase in free energy would increase the generative model's uncertainty signaling allostatic overload (Peters et al., 2017). The latter will trigger interoceptive predictions of a stress response and, should the stress become chronic, underweighting of iPEs (Figures 1Dp,Dw). Underweighting of iPEs would then generate an increase in the iSE's precision.

Interoceptive insensitivity in depression is noted in the emotion context insensitivity theory of depression (Rottenberg et al., 2005; Rottenberg and Hindash, 2015). Interestingly, a blunted emotional reactivity in depressed people was found for both positive and negative stimuli (Bylsma et al., 2008). Such blunting is associated with an attenuated prefrontal cortex activity (Stewart et al., 2020). Taken together, these data are consistent with my hypothesis that in depression, low precision of hedonic priors (encoded mostly in the prefrontal cortex) is complemented by highly precise interoceptive allostatic priors (Figures 1Dp,Dw). Such an arrangement has the potential of minimizing the depressive generative model's cumulative free energy; as low-precision exteroceptive priors accommodate the uncertain overwhelming world, hyper-precise interoceptive priors prepare the organism for its misery (allostatic overload) which is confirmed by the active inference of low mood, sadness, and fatigue. This idea has been aptly expressed by Clark and colleagues (Clark et al., 2018, p. 2278), “major depression occurs when the brain is certain that it will encounter an uncertain environment, i.e., the world that is inherently volatile, capricious, unpredictable and uncontrollable.” Unsurprisingly, chronic unpredictable stress is a widely used animal model of depression (Katz, 1982).

In the next, withdrawal stage, the protest has run its course, and the generative model is locked in a state, where high-precision self-inefficacy depresses the precision of hedonic priors, entailing overweighted ePEs (Figure 1Dw). Such a model is further confirmed by the active inference of passivity and avoidance. Meanwhile, on the interoceptive side, hyper-precise allostatic predictions may fail validation by active inference as there is no change in the allostatic load (the world remains as hostile as before). This can result in decreased iSE's precision with the entailing increase of the free energy. One way of keeping the free energy low is for the generative model to “accept” the uncontrollable nature of the world by developing high-precision allostatic predictions of energy conservation (Figure 1Dw), which would entail a transition of interoceptive active inference from high arousal and anxiety to low arousal, apathy, and decreased metabolism. That, in turn, would restore the precision of iSE and suppress iPEs, thus decreasing the generative model's free energy and stabilizing it in the withdrawal stage of DSR. Indeed, depression is often associated with a low rate of metabolism, subjectively manifesting in such symptoms as fatigue, lethargy, psychomotor retardation, loss of appetite (DSM-5). Thyroid function, a central metabolism regulator, has been long implicated in depression (Zach and Ackerman, 1988). The withdrawal stage may be the more stable one since its generative model is congruent with chronic stress.

In the evolutionary view, the next stage of DSR is recovery. As hypothesized in iESP, once DSR “bottoms out,” i.e., the withdrawal stage is complete, that creates an opportunity for recovery. The recovery is accomplished through a reversal of the DSR dynamics (Figure 1Dr). With no additional incoming noxious stimuli, random hedonic events may shift the overall balance to a more hedonic baseline (“the pendulum swinging the other way” effect). That would generate hedonically-valenced ePEs, increasing the free energy and driving increase in the precision of hedonic priors in order to suppress these ePEs. Confirming those priors through active inference would lead to increased precision of eSE as well as activation of metabolism. Active metabolism would generate iPEs with the potential of reversing the DSR's interoceptive dynamics. Metabolic activation itself can stimulate the motivational/hedonic circuitry (Ferreira et al., 2012). Also, in the absence of new FLED events and because of the positive dynamic of hedonic priors' precision, the attentional bias may change toward hedonic events, increasing the precision of hedonically-valenced ePEs and thus driving a further increase of hedonic priors' precision. Multiple factors can arrest DSR in a state of non-recovery leading to chronic depression but their analysis is beyond this paper's scope.

Any disturbance of homeostasis leads to increased uncertainty and free energy of the generative model (Peters et al., 2017), which happens due to increased iPEs. This leads to active interoceptive inference instantiated in anxiety (Paulus and Stein, 2006). In this view, any stress response starts with rising anxiety. As mentioned above, ASR is believed to be associated with hyper-precise exteroceptive and interoceptive priors (Figure 1B), and so is TSR only to a higher degree (Figure 1A) due to an abrupt recalibration of threat priors in response to trauma (Krupnik, 2020b). Thus, on the precision-weighting dysregulation continuum, ASR takes place between DSR and TSR (Figure 1). Accordingly, the dynamics of the protest stage of depression (Figure 1Dp) are similar to ASR (Figure 1B). Once initiated, ASR can lead to several outcomes. One is resolution of the allostatic load and return to the initial homeostatic state. If this fails, ASR may be arrested in a state of high anxiety, leading to an anxiety disorder, or may proceed to depression by decreasing the precision of exteroceptive priors and over-weighting ePEs. It can also be in a combined depressive-anxiety state, where high-precision threat priors co-exist with low-precision hedonic ones (Figure 1C).

## Evidence and Predictions

The proposed model of precision dysregulation in affective disorders (Figure 1) is built on multi-level theoretical assumptions as captured in the iESP acronym. This makes the model highly general and speculative, which is its main limitation. Whereas, the aforementioned literature on precision-weighting in psychopathology is consistent with the model, to date it has no direct empirical confirmation. Moreover, due to the model's wide scope, its empirical validation is likely to accumulate gradually through several lines of research, i.e., cognitive malfunction and its dynamics in affective disorders, clinical dynamics of depressive episodes, and effects of clinical interventions at different stages of DSR. Herein, I will only focus on the model's central tenet that DSR proceeds through transition from anxiety to depression (i.e., from the protest to withdrawal stage), which is associated with reduced precision of hedonic priors, and that such transition may be driven by decreasing precision of the self-efficacy meta-prior and its inversion into hyper-precise self-inefficacy (Figures 1Dp–Dw).

Several studies have reported that transition from anxiety to depression occurs more often than vice versa (ter Meulen et al., 2021), suggesting that anxiety to depression is the dominant depressive dynamic. An immediate effect of SSRI antidepressants on the amygdala (Murphy et al., 2018) also points at decreased anxiety as a probable early step in the evolution of a depressive episode. Concerning precision, anhedonia, loss of motivation and appetite are core symptoms of depression (DSM-5) consistent with hypo-precise hedonic priors. Tested in the monetary incentive delay task, depressed people show reduced gain vs. non-gain discrimination which can be interpreted as hypo-precision of hedonic priors; they also show an increased anterior cingulate activity during gain anticipation which can be interpreted as overweighted prediction errors (Knutson et al., 2008).

Perhaps most germane evidence can be gleaned from ketamine studies, since ketamine is the only known medication with an acute antidepressant effect (Jelen et al., 2020). In addition to its anti-depressant effect, ketamine has an acute anxiolytic effect (Glue et al., 2018). Among several suggested mechanisms of action, one of the better documented is ketamine's inhibition of NMDA receptors on cortical GABAergic interneurons, which leads to a surge of glutamatergic activity in the pyramidal neurons (Jelen et al., 2020). High pyramidal activity can be interpreted as activation of prediction error processing (Bastos et al., 2012), which may indicate increased precision of PEs/decreased precision of priors, as stipulated by the model (Figure 1Dw). Furthermore, some studies indicate that the antidepressant effect of ketamine is mediated by opioid receptors (Williams et al., 2018). Activation of the mu opioid receptor in the anterior insula has been suggested to sustain the hedonic tone and thus buffer negative affect in depressed people (Lutz et al., 2021). Together, these data may mean that ketamine's antidepressant action involves decreased precision of priors in the context of increased hedonic tone. If true, iESP model may explain the transient nature of ketamine effect. According to the model, recovery requires restoration of hedonic priors' precision (Figure 1Dr), which means that an increased hedonic tone alone is insufficient.

The proposed role of self-efficacy dynamics in DSR does not yet have empirical support despite the widespread acceptance of learned helplessness model of depression. I am unaware of data that could be interpreted as reflecting the dynamics of self-efficacy in a depressive episode. In an indirect support of the model, eSE's precision was shown to negatively correlate with the trait apathy (Hezemans et al., 2020).

The model's strongest claim that a progression to the withdrawal stage of depression is associated with decreased precision of both threat and hedonic priors would be best addressed in an animal model of learned helplessness since it allows for tracking and measuring the dynamics of such progression. Sucrose preference test (Willner et al., 1987) can be used as a behavioral readout of the precision of hedonic priors, and social avoidance test (Berton et al., 2006)–of the threat ones. The second part of my hypothesis suggesting that the above dynamics are released by decreased precision of the exteroceptive self-efficacy metaprior can be tested by tracking eSE's precision in depressed patients through its psychological correlate, learned helplessness measured psychometrically (Schroder and Ollis, 2013). In the proposed model (Figure 1), decreased eSE's precision should be an early event in the protest-to-withdrawal transition. Perhaps, the most immediate utility of the model is in its application to practice.

## iESP-informed Therapy for Depression

The difference between DSR, ASR, TSR, and their respective pathologies is that in pathology, the organism is arrested in a perpetual stress response cycle unable to return to optimal homeostasis. Therefore, I see the task of therapy as two-fold: first, to destabilize the generative model trapped in a stress response cycle and then to help it on a trajectory to its optimal (or at least functional) homeostatic state.

A universal approach in therapies targeting trauma and anxiety is to expose patients to their fears and avoided triggers, both external and internal such as memories and high arousal states. The utility of such an approach for depression is questionable since depressed people self-expose to disturbing memories and thoughts through rumination which is a hallmark of depression (Nolen-Hoeksema et al., 2008). Despite the widespread use of CBT, recent meta-analyses have failed to identify a superior psychotherapy for depression (Cuijpers et al., 2008). Also, the effectiveness of psychotherapy for depression has been revised down to the effect size of 0.2–0.4, i.e., in the small range (Cuijpers et al., 2019). This brings to the fore the question of developing more effective therapies.

iESP model of depression may provide guidance for its treatment. As the first, exploratory phase, it suggests that patients be assessed for the stage of their DSR, i.e., protest, withdrawal, or a budding recovery. Such an assessment would estimate the dynamics of priors' precision: eSE, iSE, hedonic, and threat. Since instruments to measure these variables directly have not yet been developed, they can be assessed through proxy measures. eSE dynamics can be estimated with the aforementioned learned helplessness scale (Schroder and Ollis, 2013). A proxy measure for hedonic priors could be Snaith–Hamilton Pleasure Scale (Snaith et al., 2018). iSE and allostatic priors' dynamics could be estimated through sympathetic and parasympathetic activity which can be considered as active inference confirming allostatic predictions. It can be tracked with wearable devices as in, e.g., Jacobson et al. (2021). Whereas, none of these measures directly addresses the precision of corresponding priors, their dynamics can aid clinicians in diagnosing the DSR stage. In addition, clinically, the withdrawal stage is characterized by low energy, apathy, and relatively low anxiety. A nascent recovery, however, may mirror the protest stage in manifesting a relative (to withdrawal) increase in energy and anxiety. The history of onset and dynamics of a depressive episode can help differentiate between these stages. Once the DSR stage is identified, appropriate interventions can be chosen.

The protest stage presents a dichotomous choice between attempting a reversal of the DSR to recovery (Figures 1Dp–Dr) and facilitating its progression to the withdrawal stage (Figures 1Dp–Dw). On the exteroceptive side, in either case, one of the objectives is to decrease the precision of the hedonic priors. Cognitive restructuring, as practiced in cognitive (Beck et al., 1979) or interpersonal (Weissman et al., 2008) therapy, is meant to achieve the protest to recovery transition. The protest to withdrawal transition is different in that it happens through a decrease of the eSE precision and its inversion into hyper-precise self-inefficacy (Figures 1Dp–Dw), for which acceptance-based interventions may be indicated. A recently developed therapy (Krupnik, 2014, 2018a) was designed on this premise. On the interoceptive side, the difference between these strategies is that protest-to-recovery goes from hyper-precise to normal energy mobilizing allostatic priors with the consequent normalization of arousal, whereas protest-to-withdrawal proceeds to hyper-precise energy conserving priors, i.e., hypo-arousal. Accordingly, protest-to-recovery can be aided by indirectly targeting arousal through decreasing the precision of threat priors with anxiolytic agents, e.g., antidepressants with a strong anxiolytic effect, as well as behaviorally through mindfulness techniques. The protest to withdrawal transition may be aided by a more direct targeting of arousal, e.g., with sympatholytic agents. The choice between protest-to-recovery vs. protest-to-withdrawal may depend on how resistant to change in each direction the protest stage is in a particular case.

Patients at the withdrawal stage require a withdrawal to recovery transition (Figures 1Dw–Dr). Interventions proposed for this task (Krupnik, 2014) are meant to stimulate the goal-oriented activity in order to increase the precision of both eSE and hedonic priors. The ensuing activation of metabolism is expected to result in an increased arousal and normalization of energy-mobilizing allostatic priors. This can be aided by behavioral activation (Jacobson et al., 2001) and opioid stimulation, e.g., by the aforementioned ketamine. The hypothesized advantage of the protest-withdrawal-recovery pathway over the protest-recovery one is that achieving acceptance of the FLED event at the withdrawal stage may make a reversal to the protest stage less likely. Given the heterogeneity and complexity of DSR manifestations and dynamics, it is unlikely that DSR stages have a hard demarcation, which may present clinicians with the challenge of a moving and blurry target. Accurate assessment of the case's dynamics and clinical judgment may assist in the choice of interventions. Overall, taking into account DSR dynamics calls for a flexible heuristic-driven (as opposed to prescriptive and manual-driven) approach to treatment that I have advocated before (Krupnik, 2018b, 2019a).

The challenge presented by the complexity of DSR dynamics and manifestations may have contributed to the aforementioned lack of progress in therapy outcome for the last 30 years. Understanding how different stages of DSR respond to treatment is, at this time, a blind spot in research on treatment of depressive disorders. Filling this gap and making therapy dynamic- and case-specific may further the development of more effective treatments.

## Author Contributions

The author confirms being the sole contributor of this work and has approved it for publication.

## Conflict of Interest

The author declares that the research was conducted in the absence of any commercial or financial relationships that could be construed as a potential conflict of interest.

## Footnotes

^1^This subdivision is similar to McEwen's model of “good,” “tolerable,” and “toxic” stress (McEwen and Gianaros, 2011), although that model is based on a different theoretical premise.

^2^In PP, emotions are regarded as interoceptive (active) inference (Seth et al., 2012).

^3^The range of allostasis and allostatic load were proposed as the axes determining the continuum of stress response (Krupnik, 2020b).
